# 3-Chloro-*N*′-[(2-meth­oxy­naphthalen-1-yl)methyl­idene]benzohydrazide

**DOI:** 10.1107/S1600536811000924

**Published:** 2011-01-12

**Authors:** Tian-Yi Li, Yan-Xia Ge

**Affiliations:** a School of Chemical Engineering, Changchun University of Technology, Changchun 130012, People’s Republic of China

## Abstract

The title compound, C_19_H_15_ClN_2_O_2_, was prepared by the reaction of 2-meth­oxy-1-naphthaldehyde with 3-chloro­benzohydrazide in methanol. The dihedral angle between the benzene ring and the naphthyl ring system is 69.0 (3)°. In the crystal, inter­molecular N—H⋯O hydrogen bonds link the mol­ecules into chains along the *c* axis. The crystal packing exhibits π–π inter­actions, as indicated by distances of 3.768 (3) Å between the centroids of the naphthyl rings of neighbouring mol­ecules.

## Related literature

For a related structure, see: Li & Li (2011) ▶. For reference bond lengths, see: Allen *et al.* (1987 ▶). For details of the synthesis, see: Zhu (2010 ▶).
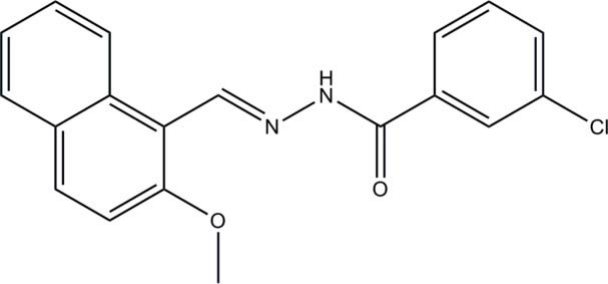


## Experimental

### 

#### Crystal data

C_19_H_15_ClN_2_O_2_
*M* *_r_* = 338.78
Monoclinic, 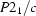

*a* = 12.181 (2) Å
*b* = 16.953 (4) Å
*c* = 8.5482 (15) Å
β = 109.446 (2)°
*V* = 1664.5 (6) Å^3^
*Z* = 4
Mo *K*α radiation
μ = 0.24 mm^−1^
*T* = 298 K
0.18 × 0.18 × 0.16 mm

#### Data collection

Bruker APEXII CCD area-detector diffractometer
Absorption correction: multi-scan (*SADABS*; Bruker, 2005 ▶) *T*_min_ = 0.958, *T*_max_ = 0.962
8964 measured reflections
3586 independent reflections
1624 reflections with *I* > 2σ(*I*)
*R*_int_ = 0.067

#### Refinement

*R*[*F* ^2^ > 2σ(*F* ^2^)] = 0.060
*wR*(*F* ^2^) = 0.147
*S* = 1.00
3586 reflections
221 parameters
1 restraint
H atoms treated by a mixture of independent and constrained refinement
Δρ_max_ = 0.19 e Å^−3^
Δρ_min_ = −0.24 e Å^−3^

### 

Data collection: *APEX2* (Bruker, 2005 ▶); cell refinement: *SAINT* (Bruker, 2005 ▶); data reduction: *SAINT*; program(s) used to solve structure: *SHELXTL* (Sheldrick, 2008 ▶); program(s) used to refine structure: *SHELXTL*; molecular graphics: *SHELXTL*; software used to prepare material for publication: *SHELXTL*.

## Supplementary Material

Crystal structure: contains datablocks global, I. DOI: 10.1107/S1600536811000924/cv5034sup1.cif

Structure factors: contains datablocks I. DOI: 10.1107/S1600536811000924/cv5034Isup2.hkl

Additional supplementary materials:  crystallographic information; 3D view; checkCIF report

## Figures and Tables

**Table 1:** Hydrogen-bond geometry (Å, °)

*D*—H⋯*A*	*D*—H	H⋯*A*	*D*⋯*A*	*D*—H⋯*A*
N2—H2⋯O2^i^	0.91 (3)	2.04 (3)	2.937 (3)	170 (3)

Symmetry code: (i) 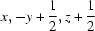.

**Fig. 1.**
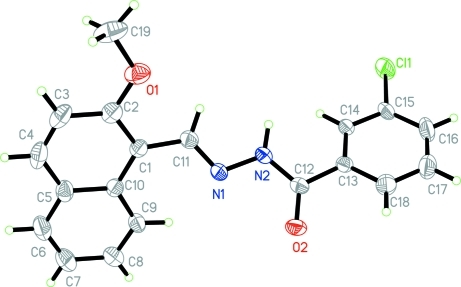
The molecular structure of the title compound with 30% probability displacement ellipsoids for non-hydrogen atoms.

**Fig. 2.**
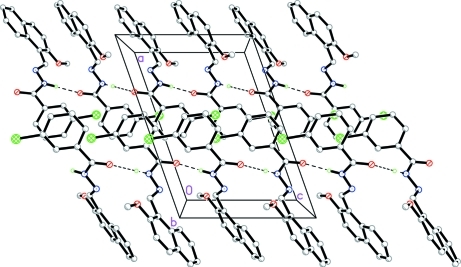
A portion of the crystal packing showing hydrogen bonds as dashed lines.

## supplementary crystallographic information

### Comment

In continuation of our structural study of naphthylidenebenzohydrazide derivatives (Li & Li, 2011) we present here the title compound (I).

In (I) (Fig. 1),the dihedral angle between the benzene ring and the naphthyl bicycle is 69.0 (3)°. All the bond lengths are within normal values (Allen *et al.*, 1987). Intermolecular N—H···O hydrogen bonds (Table 1) link the molecules into chains along the *c* axis (Fig. 2). The crystal packing exhibits π-π interactions proved by short distances of 3.768 (3) Å between the centroids of naphthyl rings from the neighbouring molecules.

### Experimental

The compound was prepared and crystallized according to the literature method (Zhu, 2010). 2-Methoxy-1-naphthaldehyde (0.186 g, 1 mmol) and 3-chlorobenzohydrazide (0.171 g, 1 mmol) were dissolved in 30 ml absolute methanol. The mixture was stirred at reflux for 10 min, and cooled to room temperature. The clear colorless solution was left to slow evaporation in air for eight days, yielding colorless block-shaped crystals, which were collected by filtration and washed with methanol.

### Refinement

The amino H atom was located from a difference Fourier map and refined isotropically, with the N—H distance restrained to 0.90 (1) Å. The other H atoms were positioned geometrically and refined using the riding-model approximation, with C—H = 0.93–0.96 Å, and *U*_iso_(H) = 1.2*U*_eq_(C) or *U*_iso_(H) = 1.5*U*_eq_(C19).

### Crystal data

**Table:**

C_19_H_15_ClN_2_O_2_	*F*(000) = 704
*M**_r_* = 338.78	*D*_x_ = 1.352 Mg m^−^^3^
Monoclinic, *P*2_1_/*c*	Mo *K*α radiation, λ = 0.71073 Å
*a* = 12.181 (2) Å	Cell parameters from 762 reflections
*b* = 16.953 (4) Å	θ = 2.5–24.3°
*c* = 8.5482 (15) Å	µ = 0.24 mm^−^^1^
β = 109.446 (2)°	*T* = 298 K
*V* = 1664.5 (6) Å^3^	Block, colourless
*Z* = 4	0.18 × 0.18 × 0.16 mm

### Data collection

**Table:**

Bruker APEXII CCD area-detector diffractometer	3586 independent reflections
Radiation source: fine-focus sealed tube	1624 reflections with *I* > 2σ(*I*)
graphite	*R*_int_ = 0.067
ω scans	θ_max_ = 27.0°, θ_min_ = 2.4°
Absorption correction: multi-scan (*SADABS*; Bruker, 2005)	*h* = −14→15
*T*_min_ = 0.958, *T*_max_ = 0.962	*k* = −19→21
8964 measured reflections	*l* = −10→10

### Refinement

**Table:**

Refinement on *F*^2^	Primary atom site location: structure-invariant direct methods
Least-squares matrix: full	Secondary atom site location: difference Fourier map
*R*[*F*^2^ > 2σ(*F*^2^)] = 0.060	Hydrogen site location: inferred from neighbouring sites
*wR*(*F*^2^) = 0.147	H atoms treated by a mixture of independent and constrained refinement
*S* = 1.00	*w* = 1/[σ^2^(*F*_o_^2^) + (0.0386*P*)^2^] where *P* = (*F*_o_^2^ + 2*F*_c_^2^)/3
3586 reflections	(Δ/σ)_max_ < 0.001
221 parameters	Δρ_max_ = 0.19 e Å^−^^3^
1 restraint	Δρ_min_ = −0.24 e Å^−^^3^

### Special details

**Table:**

Geometry. All e.s.d.'s (except the e.s.d. in the dihedral angle between two l.s. planes) are estimated using the full covariance matrix. The cell e.s.d.'s are taken into account individually in the estimation of e.s.d.'s in distances, angles and torsion angles; correlations between e.s.d.'s in cell parameters are only used when they are defined by crystal symmetry. An approximate (isotropic) treatment of cell e.s.d.'s is used for estimating e.s.d.'s involving l.s. planes.
Refinement. Refinement of *F*^2^ against ALL reflections. The weighted *R*-factor *wR* and goodness of fit *S* are based on *F*^2^, conventional *R*-factors *R* are based on *F*, with *F* set to zero for negative *F*^2^. The threshold expression of *F*^2^ > σ(*F*^2^) is used only for calculating *R*-factors(gt) *etc*. and is not relevant to the choice of reflections for refinement. *R*-factors based on *F*^2^ are statistically about twice as large as those based on *F*, and *R*- factors based on ALL data will be even larger.

### Fractional atomic coordinates and isotropic or equivalent isotropic displacement parameters (Å^2^)

**Table:**

	*x*	*y*	*z*	*U*_iso_*/*U*_eq_	
Cl1	0.57105 (8)	0.10288 (6)	1.03193 (12)	0.0793 (4)	
N1	0.8646 (2)	0.28050 (15)	0.6055 (3)	0.0469 (7)	
N2	0.7753 (2)	0.24546 (15)	0.6487 (3)	0.0467 (7)	
O1	0.9252 (2)	0.49223 (13)	0.7763 (3)	0.0709 (7)	
O2	0.71701 (19)	0.17210 (12)	0.4129 (3)	0.0619 (7)	
C1	1.0045 (3)	0.38640 (17)	0.6734 (4)	0.0453 (8)	
C2	1.0122 (3)	0.46507 (19)	0.7236 (4)	0.0540 (9)	
C3	1.1046 (3)	0.5131 (2)	0.7182 (4)	0.0707 (11)	
H3	1.1088	0.5653	0.7527	0.085*	
C4	1.1874 (3)	0.4831 (2)	0.6626 (5)	0.0753 (11)	
H4	1.2479	0.5157	0.6589	0.090*	
C5	1.1857 (3)	0.4049 (2)	0.6107 (4)	0.0563 (9)	
C6	1.2739 (3)	0.3739 (2)	0.5573 (5)	0.0737 (11)	
H6	1.3347	0.4067	0.5556	0.088*	
C7	1.2739 (3)	0.2977 (3)	0.5082 (5)	0.0751 (11)	
H7	1.3337	0.2785	0.4739	0.090*	
C8	1.1819 (3)	0.2489 (2)	0.5103 (4)	0.0676 (10)	
H8	1.1807	0.1965	0.4773	0.081*	
C9	1.0942 (3)	0.27685 (19)	0.5598 (4)	0.0549 (9)	
H9	1.0337	0.2431	0.5582	0.066*	
C10	1.0917 (3)	0.35562 (17)	0.6136 (4)	0.0458 (8)	
C11	0.9075 (3)	0.34138 (18)	0.6916 (4)	0.0488 (8)	
H11	0.8752	0.3580	0.7703	0.059*	
C12	0.7072 (3)	0.19108 (18)	0.5452 (4)	0.0463 (8)	
C13	0.6157 (3)	0.15628 (16)	0.6049 (4)	0.0418 (7)	
C14	0.6300 (2)	0.14888 (16)	0.7713 (4)	0.0433 (8)	
H14	0.6972	0.1682	0.8504	0.052*	
C15	0.5463 (3)	0.11327 (18)	0.8211 (4)	0.0506 (8)	
C16	0.4458 (3)	0.0859 (2)	0.7070 (5)	0.0752 (11)	
H16	0.3886	0.0626	0.7417	0.090*	
C17	0.4298 (3)	0.0930 (2)	0.5414 (5)	0.0840 (12)	
H17	0.3618	0.0743	0.4633	0.101*	
C18	0.5144 (3)	0.1278 (2)	0.4901 (4)	0.0670 (10)	
H18	0.5031	0.1322	0.3774	0.080*	
C19	0.9307 (4)	0.5719 (2)	0.8359 (5)	0.0905 (13)	
H19A	1.0029	0.5798	0.9245	0.136*	
H19B	0.8669	0.5812	0.8758	0.136*	
H19C	0.9260	0.6079	0.7471	0.136*	
H2	0.757 (3)	0.265 (2)	0.736 (3)	0.109*	

### Atomic displacement parameters (Å^2^)

**Table:**

	*U*^11^	*U*^22^	*U*^33^	*U*^12^	*U*^13^	*U*^23^
Cl1	0.0826 (7)	0.1061 (8)	0.0635 (7)	−0.0240 (6)	0.0433 (6)	0.0039 (5)
N1	0.0513 (16)	0.0548 (16)	0.0406 (16)	−0.0094 (13)	0.0231 (14)	−0.0007 (13)
N2	0.0505 (16)	0.0514 (16)	0.0461 (18)	−0.0066 (13)	0.0267 (15)	−0.0026 (13)
O1	0.0942 (18)	0.0526 (15)	0.0704 (18)	0.0061 (13)	0.0333 (16)	−0.0040 (12)
O2	0.0833 (17)	0.0664 (15)	0.0490 (15)	−0.0153 (12)	0.0392 (14)	−0.0103 (11)
C1	0.055 (2)	0.0434 (19)	0.0354 (19)	−0.0063 (15)	0.0130 (17)	0.0031 (14)
C2	0.066 (2)	0.050 (2)	0.041 (2)	−0.0017 (18)	0.0120 (18)	0.0071 (16)
C3	0.087 (3)	0.047 (2)	0.067 (3)	−0.015 (2)	0.010 (2)	−0.0055 (18)
C4	0.070 (3)	0.067 (3)	0.081 (3)	−0.030 (2)	0.015 (2)	0.002 (2)
C5	0.054 (2)	0.065 (2)	0.047 (2)	−0.0132 (17)	0.0121 (18)	0.0083 (18)
C6	0.055 (2)	0.091 (3)	0.080 (3)	−0.014 (2)	0.029 (2)	0.008 (2)
C7	0.056 (2)	0.094 (3)	0.085 (3)	0.004 (2)	0.037 (2)	0.006 (2)
C8	0.068 (2)	0.075 (2)	0.068 (3)	−0.001 (2)	0.034 (2)	−0.001 (2)
C9	0.051 (2)	0.064 (2)	0.054 (2)	−0.0092 (17)	0.0235 (19)	0.0010 (17)
C10	0.053 (2)	0.0466 (19)	0.0367 (19)	−0.0077 (15)	0.0138 (17)	0.0048 (15)
C11	0.052 (2)	0.055 (2)	0.042 (2)	−0.0037 (16)	0.0195 (17)	0.0020 (17)
C12	0.053 (2)	0.0482 (19)	0.044 (2)	0.0026 (16)	0.0239 (18)	0.0015 (16)
C13	0.0424 (19)	0.0426 (17)	0.042 (2)	0.0016 (14)	0.0169 (17)	−0.0018 (15)
C14	0.0440 (18)	0.0436 (17)	0.047 (2)	−0.0031 (14)	0.0221 (17)	−0.0027 (15)
C15	0.048 (2)	0.058 (2)	0.055 (2)	−0.0020 (16)	0.0298 (19)	0.0024 (17)
C16	0.049 (2)	0.098 (3)	0.086 (3)	−0.019 (2)	0.032 (2)	0.003 (2)
C17	0.055 (2)	0.126 (4)	0.065 (3)	−0.029 (2)	0.012 (2)	−0.007 (3)
C18	0.064 (2)	0.087 (3)	0.049 (2)	−0.010 (2)	0.018 (2)	0.0005 (19)
C19	0.141 (4)	0.059 (2)	0.065 (3)	0.024 (2)	0.025 (3)	−0.007 (2)

### Geometric parameters (Å, °)

**Table:**

Cl1—C15	1.735 (3)	C7—H7	0.9300
N1—C11	1.274 (3)	C8—C9	1.358 (4)
N1—N2	1.392 (3)	C8—H8	0.9300
N2—C12	1.354 (4)	C9—C10	1.416 (4)
N2—H2	0.91 (3)	C9—H9	0.9300
O1—C2	1.362 (4)	C11—H11	0.9300
O1—C19	1.437 (4)	C12—C13	1.494 (4)
O2—C12	1.219 (3)	C13—C14	1.380 (4)
C1—C2	1.394 (4)	C13—C18	1.383 (4)
C1—C10	1.422 (4)	C14—C15	1.370 (4)
C1—C11	1.458 (4)	C14—H14	0.9300
C2—C3	1.402 (4)	C15—C16	1.367 (4)
C3—C4	1.349 (4)	C16—C17	1.368 (5)
C3—H3	0.9300	C16—H16	0.9300
C4—C5	1.395 (5)	C17—C18	1.379 (5)
C4—H4	0.9300	C17—H17	0.9300
C5—C6	1.402 (5)	C18—H18	0.9300
C5—C10	1.425 (4)	C19—H19A	0.9600
C6—C7	1.358 (5)	C19—H19B	0.9600
C6—H6	0.9300	C19—H19C	0.9600
C7—C8	1.399 (5)		
			
C11—N1—N2	113.5 (2)	C9—C10—C5	116.3 (3)
C12—N2—N1	118.7 (2)	C1—C10—C5	119.5 (3)
C12—N2—H2	121 (2)	N1—C11—C1	123.7 (3)
N1—N2—H2	119 (2)	N1—C11—H11	118.2
C2—O1—C19	119.1 (3)	C1—C11—H11	118.2
C2—C1—C10	118.7 (3)	O2—C12—N2	123.9 (3)
C2—C1—C11	116.2 (3)	O2—C12—C13	121.8 (3)
C10—C1—C11	125.1 (3)	N2—C12—C13	114.2 (3)
O1—C2—C1	116.4 (3)	C14—C13—C18	118.5 (3)
O1—C2—C3	122.4 (3)	C14—C13—C12	122.4 (3)
C1—C2—C3	121.1 (3)	C18—C13—C12	119.1 (3)
C4—C3—C2	119.7 (3)	C15—C14—C13	120.6 (3)
C4—C3—H3	120.1	C15—C14—H14	119.7
C2—C3—H3	120.1	C13—C14—H14	119.7
C3—C4—C5	122.4 (3)	C16—C15—C14	120.7 (3)
C3—C4—H4	118.8	C16—C15—Cl1	120.8 (2)
C5—C4—H4	118.8	C14—C15—Cl1	118.5 (3)
C4—C5—C6	121.9 (3)	C15—C16—C17	119.5 (3)
C4—C5—C10	118.6 (3)	C15—C16—H16	120.2
C6—C5—C10	119.5 (3)	C17—C16—H16	120.2
C7—C6—C5	122.4 (3)	C16—C17—C18	120.2 (3)
C7—C6—H6	118.8	C16—C17—H17	119.9
C5—C6—H6	118.8	C18—C17—H17	119.9
C6—C7—C8	118.4 (3)	C17—C18—C13	120.5 (3)
C6—C7—H7	120.8	C17—C18—H18	119.7
C8—C7—H7	120.8	C13—C18—H18	119.7
C9—C8—C7	121.0 (3)	O1—C19—H19A	109.5
C9—C8—H8	119.5	O1—C19—H19B	109.5
C7—C8—H8	119.5	H19A—C19—H19B	109.5
C8—C9—C10	122.3 (3)	O1—C19—H19C	109.5
C8—C9—H9	118.9	H19A—C19—H19C	109.5
C10—C9—H9	118.9	H19B—C19—H19C	109.5
C9—C10—C1	124.2 (3)		

### Hydrogen-bond geometry (Å, °)

**Table:**

*D*—H···*A*	*D*—H	H···*A*	*D*···*A*	*D*—H···*A*
N2—H2···O2^i^	0.91 (3)	2.04 (3)	2.937 (3)	170 (3)

Symmetry codes: (i) *x*, −*y*+1/2, *z*+1/2.
